# Editorial Comment to Progressive plasmacytoid variant bladder cancer with retroperitoneal dissemination: An autopsy case report

**DOI:** 10.1002/iju5.12183

**Published:** 2020-06-28

**Authors:** Tetsutaro Hayashi, Jun Teishima

**Affiliations:** ^1^ Department of Urology Institute of Biomedical and Health Sciences Integrated Health Sciences Hiroshima University Hiroshima Japan

Kohada *et al.* reported a case of plasmacytoid urothelial carcinoma (PUC), an aggressive variant of urothelial carcinoma (UC).^1^ The authors studied the immunohistochemical (IHC) characterization of PUC. Since its optimal therapy has not yet been established, these data provide important insight into novel treatment of PUC.

As the authors described, the sheet‐like growth pattern of PUC makes disease spread during the early stages much more difficult to capture on cross‐sectional imaging. Therefore, urologists should be cognizant of the inherent limitations in diagnosing PUC relapse. Patients may require treatment based on symptoms and clinical suspicion.^2^ Circulating tumor cells (CTCs) are indicators of recurrence and prognosis for several types of cancers, and can provide information about tumor biology. The authors identified two CTCs after first‐line treatment and IHC analysis of CTCs showed programmed death ligand 1 expression. This information was useful for determining an appropriate second‐line treatment. Although further studies are needed, in the future CTCs might serve as alternative forms of surveillance and help determine the optimal treatment strategy for PUC.

Autopsies can establish the extent of clinical disease and help clinicians to understand the clinical and pathological aspects of that disease.^2^ During IHC analysis, almost all PUC cases showed expression of urothelial markers (GATA‐3) and plasma cell markers (CD138). Half of the PUC cases showed loss of E‐cadherin expression. The IHC analysis of pathological specimens can distinguish PUC from lobular breast and diffuse gastric carcinomas which share a similar morphologic appearance.^2^, ^3^ In this case, the authors revealed higher HER2 expression within the autopsy PUC specimen. Kim *et al*. reported that PUC frequently showed HER2 protein overexpression and HER2 gene amplification.^4^ Al‐Ahmadie *et al.* also reported that ERBB2 was frequently observed among clinically actionable alterations in whole‐exome sequencing of PUC.^3^ HER2 may be a good candidate for targeted PUC therapy. We have previously reported that HER2 antibody–drug conjugates (ADCs) have promising anti‐tumor effects in preclinical models of HER2‐overexpressing UC compared with the HER2 antibody alone.^5^ Recent genomic studies suggest that UC of the bladder, especially the luminal subtype of UC, could potentially respond to HER2‐targeted therapy. Therefore, novel and potent HER2‐ADCs should be evaluated in future clinical trials. The molecular characterization of PUC opens the door to target therapies, which might improve the prognosis for patients with this rare and aggressive variant of UC.

## Conflict of interest

The authors declare no conflict of interest.
